# Commonly used C‐peptide assays show differing associations with CGM metrics

**DOI:** 10.1111/dom.70351

**Published:** 2025-12-04

**Authors:** Catriona Clarke, Mark W. J. Strachan, Fraser W. Gibb

**Affiliations:** ^1^ Department of Clinical Biochemistry NHS Lothian Edinburgh UK; ^2^ Edinburgh Centre for Endocrinology and Diabetes, NHS Lothian Edinburgh UK; ^3^ University/BHF Centre for Cardiovascular Science, Queen's Medical Research Institute, University of Edinburgh Edinburgh UK

## 1 BACKGROUND

C‐peptide persistence is associated with favourable and clinically important differences in continuous glucose monitoring (CGM) metrics and HbA1c.^1^, ^2^, ^3^ We have previously shown that random C‐peptide around 50pM is also an important clinical endpoint in studies assessing beta cell preservation.^4^ However, C‐peptide assays have not been standardised and consequently there is substantial variability between assays, which may limit the comparability of studies reporting C‐peptide results.^5^, ^6^ We sought to compare whether results from two commonly used assays are associated with differences in glycaemic metrics.

## 2 METHODS

Our centre routinely assesses random C‐peptide in individuals with a diagnosis of type 1 diabetes to identify potential misclassification.^7^ In October 2021 we switched from the Abbott Architect immunoassay to the Roche immunoassay on the Cobas e801 platform. A numeric result is not provided by our laboratory for results <50pM, as this corresponds to the manufacturer's recommended limit of quantification. We have previously reported the relationship between these assays: Abbott C‐peptide (pM) = 0.8755 × Roche C‐peptide (pM) − 40.6.^7^ For the current analysis, we identified 290 individuals with C‐peptide measured by the Abbott assay and matched them with 290 individuals where C‐peptide was measured using the Roche assay (from a pool of 945 individuals^2^) by propensity‐score matching accounting for age, sex, smoking status, BMI, diabetes duration, time in range, time below range and time above range. All glucose metrics were derived from Freestyle Libre 2 and reflect a 2‐week period corresponding with C‐peptide measurement. CGM metrics and targets are reported in line with the CGM international consensus document.^8^ As a service evaluation of routinely collected data, this project did not require ethical approval. Results are presented as median (interquartile range). Unpaired data were compared using Wilcoxon rank‐sum test. Categorical data were compared using chi‐squared tests. *p* values <0.05 were taken to indicate statistical significance. Statistical analyses were performed using R Studio (version 2023.12.1).

## 3 RESULTS

**FIGURE 1:**
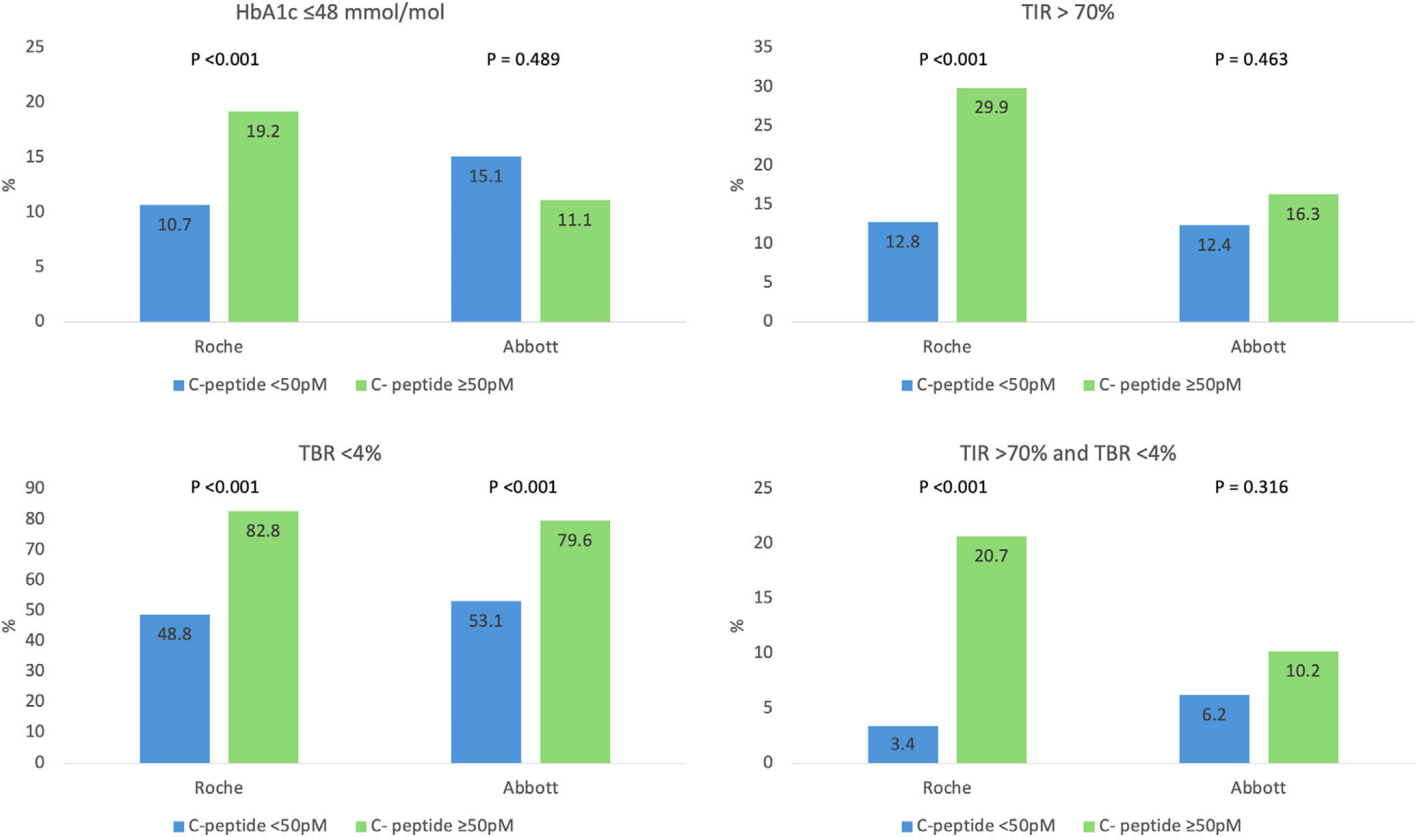
Percentage of individuals meeting HbA1c and continuous glucose monitoring consensus targets with C‐peptide <50 and ≥ 50pM in the Abbott assay and Roche assay cohorts. TBR, time below range; TIR, time in range.

Median age was 42 years (30–53) and 53% of participants were male. Full cohort characteristics are presented in Table S1. Thirty percent of results with the Roche assay were ≥ 50pM compared to 17% with the Abbott assay (*p* < 0.001). Differences in glucose metrics, stratified by detectable C‐peptide, are presented for both the Roche and Abbott assays in Table 1. Detectable C‐peptide was associated with higher time in range and lower time > 13.9 mM with the Roche assay but not the Abbott assay. Detectable C‐peptide was associated with lower time below range and coefficient of variation for glucose in both the Roche and Abbott assays. Average glucose and HbA1c were not lower in those with detectable C‐peptide by either assay. Associations with C‐peptide status and meeting CGM and HbA1c targets are presented in Figure 1.

**TABLE 1:** Comparison of glucose metrics in those with C‐peptide <50pM and ≥ 50pM in the Abbott assay and Roche assay cohorts.

	Abbott assay			Roche assay		
	C‐peptide <50 pM	C‐peptide ≥50 pM	*p*	C‐peptide <50pM	C‐peptide ≥50pM	*p*
HbA1c (mmol/mol)	60 (52–68)	64 (54–69)	0.337	62 (54–72)	62 (51–74)	0.949
Time in range (%)	52 (40–64)	49 (36–63)	0.660	47 (35–62)	54 (35–74)	0.048
Time below range (%)	3 (1–7)	1 (0–3)	<0.001	4 (1–7)	1 (0–3)	<0.001
Time above range (%)	43 (28–57)	49 (31–62)	0.370	48 (33–61)	46 (22–64)	0.238
Time > 13.9 mM (%)	13 (5–25)	12 (3–22)	0.306	18 (8–31)	11 (3–30)	0.020
Coefficient of variation for glucose (%)	38.9 (33.9–43.7)	31.5 (27.9–37.6)	<0.001	39.3 (35.6–44.4)	32.3 (29.0–36.8)	<0.001
Mean glucose (mM)	9.8 (8.4–11.2)	10.2 (8.7–11.1)	0.607	10.1 (8.8–11.9)	10.0 (7.9–11.5)	0.173

## 4 DISCUSSION

We have shown that different C‐peptide assays have different attributes with respect to their associations with glycaemic metrics. The Roche assay has been shown to have a mean bias ranging from +13.3% to +36.6% in relation to other commonly used assays.^5^ It is highly likely, therefore, that the clinically relevant C‐peptide threshold for the Abbott assay is lower than 50 pM derived from data using the Roche assay. Assay bias of this magnitude is likely to result in misclassification of many individuals when using any fixed threshold, as attested to by the almost twofold higher prevalence of C‐peptide ≥50pM in the Roche group. These data confirm that C‐peptide is related to CGM outcomes, especially hypoglycaemia, in people with type 1 diabetes but suggest that more effective standardisation of C‐peptide assays is required to improve clinical care and the generalisability of clinical studies.

## CONFLICT OF INTEREST STATEMENT

FWG has received advisory board fees from Abbott Diabetes Care and Roche.

## Supporting information


**Table S1.** Clinical and biochemical characteristics of the study cohorts. P value refers to the comparison between the Abbott and Roche assay groups.

## Data Availability

The data that support the findings of this study are available on request from the corresponding author. The data are not publicly available due to privacy or ethical restrictions.
