# Supplementary material for: Commonly used C‐peptide assays show differing associations with CGM metrics
Source: Diabetes Obes Metab. 2025 Dec 4;28(3):2422–4. doi: 10.1111/dom.70351 (PMC12890733; doi:10.1111/dom.70351)
Supplement: Supplementary file 1 — Table S1. Clinical and biochemical characteristics of the study cohorts. P value refers to the comparison between the Abbott and Roche assay groups. [file DOM-28-2422-s001.docx]

**Supplementary table 1**: Clinical and biochemical characteristics of the study cohorts. P value refers to the comparison between the Abbott and Roche assay groups.

|  | Total (n = 580) | Abbott (n = 290) | Roche (n = 290) | P |
| --- | --- | --- | --- | --- |
| Age (years) | 42 (30 – 53) | 43 (30 – 54) | 41 (30 – 53) | 0.524 |
| Diabetes duration (years) | 21 (11 – 33) | 21 (12 – 32) | 21 (10 – 34) | 0.783 |
| Sex | Male: 310  Female: 270 | Male: 154  Female: 136 | Male: 156  Female: 134 | 0.868 |
| BMI (kg/m^2^) | 27.3 (24.1 – 31.7) | 27.3 (24.0 – 31.4) | 27.5 (24.2 – 31.9) | 0.446 |
| Current smoker | Yes: 536  No: 44 | Yes: 23  No: 267 | Yes: 21  No: 269 | 0.754 |
| Scottish Index of Multiple deprivation quintile (1 – most deprived) | SIMD 1 or 2: 150  SIMD 3 to 5: 401  NA: 29 | SIMD 1 or 2: 71  SIMD 3 to 5: 205  NA: 14 | SIMD 1 or 2: 79  SIMD 3 to 5: 196  NA: 15 | 0.429 |
| C-peptide category | <50pM: 444  50 – 100pM: 37  101 – 200pM: 33  201 – 400pM: 33  >400pM: 33 | <50pM: 241  50 – 100pM: 18  101 – 200pM: 12  201 – 400pM: 11  >400pM: 8 | <50pM: 203  50 – 100pM: 19  101 – 200pM: 21  201 – 400pM: 22  >400pM: 25 | 0.001 |
| HbA1c (mmol/mol) | 61 (53 – 70) | 60 (52 – 69) | 62 (54 – 73) | 0.098 |
| Time in range (%) | 50 (37 – 64) | 51 (39 – 64) | 49 (34 – 65) | 0.283 |
| Time below range (%) | 3 (1 – 6) | 3 (1 – 6) | 3 (1 – 6) | 0.320 |
| Time above range (%) | 45 (28 – 60) | 43 (28 – 58) | 47 (28 – 61) | 0.264 |
| Time >13.9mM (%) | 14 (6 – 28) | 13 (5 – 24) | 17 (6 – 31) | 0.019 |
| CV glucose (%) | 37.6 (32.9 – 43.0) | 37.8 (32.9 – 43.0) | 37.4 (32.8 – 43.2) | 0.755 |
| Mean glucose (mM) | 9.9 (8.5 – 11.5) | 9.9 (8.4 – 11.2) | 10.0 (8.5 – 11.9) | 0.218 |
| TIR consensus target met? | Yes: 90  No: 490 | Yes: 38  No: 252 | Yes: 52  No: 238 | 0.108 |
| TBR consensus target met? | Yes: 338  No: 242 | Yes: 167  No: 123 | Yes: 171  No: 119 | 0.736 |
| Both consensus targets met? | Yes: 45  No: 535 | Yes: 20  No: 270 | Yes: 25  No: 265 | 0.437 |
| HbA1c ≤48 mmol/mol | Yes: 72  No: 450  NA: 58 | Yes: 37  No: 220  NA: 33 | Yes: 35  No: 230  NA: 25 | 0.694 |
